# The Cholera Abroad

**Published:** 1884-08

**Authors:** 


### THE CHOLERA ABROAD.

The epidemic continues to advance, and there is now little hope of preventing its extension to the larger commercial and trading centers of Europe. Already several towns between Toulon and Paris are infected, and recent reports would indicate that the disease had appeared in Paris. All reports from a French source must be received with some allowance, as they have more or less of a commercial bias. The cry, "Don't call it cholera, lest it injure trade," is but an echo of the sentiment that in 1866 censured a medical college in this city, through the columns of the public press, for sending its class home after the loss of three of their number and the president of the faculty.

That there is nothing new under the sun is particularly true of the present epidemic. Most of our domestic and foreign exchanges have considered the subject at greater or less length, but have thus far contributed to it little new or specially important.

At a recent meeting of the French Academy, M. Faurel, in his official communication, stated that the epidemic was not Asiatic cholera, but cholera nostras, due to the assembling of a large number of troops under bad hygienic conditions. This astonishing statement was made ten days after the epidemic had occurred. In closing his communication, M. Faurel assured the Academy, with characteristic French emphasis, that he had no doubt of the result (*i. e.*, the speedy subsidence of the epidemic), *mais je n'ai pas la pretention d' etre infallible*, and in the light of subsequent events there is but little doubt but that his brethren will agree with the latter statement. This discussion called forth a sharp criticism from Prof. Virchow in *Die Nation* of July 5. He arraigns the French government very severely for their inactivity in the early stages of the epidemic. One week after the first case appeared in Toulon, and while every day there was an increasing number of deaths, the French government remained strangely apathetic and no effort was made to prevent the spread of the disease. Whether it would have been possible to protect Marseilles is, in the opinion of Prof. Virchow, uncertain. But he clearly points out the failure of the French to make an early diagnosis, and their too confident reliance upon the pleasing assumption that it was only sporadic cholera. That they did not find the peculiar bacillus was no excuse for the error, as the diagnosis has been made many times before the bacillus was ever known.

Later, some physicians in Paris, who had too quickly assumed the sporadic nature of the present epidemic, announced that it was only a *mild form* of Asiatic cholera. What basis had they for the opinion that it was a mild form of the disease, or an epidemic that would soon pass away? Is there anything in the history of past epidemics that will permit us to say that in this place, at such a time, the disease will be mild and will soon disappear? We think not. And yet it was on such premises that French sanitary officials allowed themselves to be lulled into a false security. The fact remains that the cholera was introduced by a French transport ship from Tonkin, and that through the apathy or ignorance of French sanitary officials the golden opportunity of stamping out the disease in its incipiency was lost.

The discovery of the bacillus has in no sense helped us to deal with the vital and practical points of the question. The probabilities point strongly to a bacterial origin of the disease, but the crucial test of inoculating animals has not succeeded, so the theory must still be held as doubtful, or at least not proved. That the peculiar bacillus may not be found in sporadic cholera is still uncertain, and helps to throw doubt on the theory. In the meantime we are left with a crowd of these unwelcome visitors knocking at our doors; our trans-Atlantic brethren presenting us nothing better than a possibility of a delay of two weeks for finding out what is the matter; and a brilliant leading article in the London *Students' Journal and Hospital Gazette*, advising us to use the opium and lead acetate pill after we have made the correct diagnosis! Do "they do *these* things better in France?"

